# Draft genome sequence of Japanese pear (*Pyrus pyrifolia*)

**DOI:** 10.1016/j.dib.2018.07.007

**Published:** 2018-07-10

**Authors:** Yoshihiro Takemura, Fumio Tamura

**Affiliations:** Faculty of Agriculture, Tottori University, Koyama, Tottori 680–8553, Japan

## Abstract

The Japanese pear (*Pyrus pyrifolia*), a member of the family Rosaceae, is one of the most important fruit trees in Japan. This article documents the public availability of the partial draft genome sequence data of the Japanese pear strain TH3, which is the S1 of ‘Osa-Nijisseiki’ and is homozygous for the S_4_^sm^ gene. This dataset may be used to prepare molecular markers for breeding of new cultivars having a crisp texture and feel. This data will also help research on physiological disorders affecting Japanese pear fruit. We sequenced paired-end libraries using Illumina HiSeq. 2500 and generated approximately 212M reads. Data on the draft genome obtained in this study has been deposited to the DNA Data Bank of Japan (DDBJ). The read data were submitted to the DDBJ Read Archive (BioProject: PRJDB6878, BioSample: SAMD00117051).

**Specifications Table**

**Table:** 

Subject area	Biology
More specific subject area	Horticultural science, Genomics
Type of data	Genome sequence data
How data was acquired	Illumina HiSeq. 2500 Next Generation Sequencing Platforms
Data format	Raw sequencing reads
Experimental factors	Draft genome sequence of Japanese pear was performed by using Illumina HiSeq.
Experimental features	Sequencing was performed according to Illumina sequencing protocols for DNA-seq
Data source location	Shizuoka, Japan
Data accessibility	The draft genome sequences of Japanese pear (*Pyrus pyrifolia*) have been deposited in the DNA Data Bank of Japan (DDBJ) under the DDBJ Read Archive (BioProject: PRJDB6878 (https://ddbj.nig.ac.jp/BPSearch/bioproject?acc=PRJDB6878), BioSample: SAMD00117051).

**Value of the data**

● In study of evolutionary processes occurring in *Pyrus* plants, this dataset may be used to compare between the genomic data of other *Pyrus* species.
● This data will be useful to prepare molecular markers for breeding of new cultivars having a crisp texture and feel because the flesh of the Japanese pear has many stone cells.
● This data will further be valuable for more detailed study on physiological disorders affecting Japanese pear fruit.

## 1 Data

The Japanese pear, one of the most important fruit trees in Japan, is also widely distributed in China and Korea [1], where more than a thousand cultivars are grown naturally or commercially [2]. We present a partial draft genome sequence of Japanese pear strain TH3, which is the S1 of ‘Osa-Nijisseiki’ and is homozygous for the S_4_^sm^ gene [3]. We sequenced paired-end libraries using Illumina HiSeq. 2500 and generated approximately 212 M reads. The average percentage of Q30 bases (bases with a quality score of 30) and mean quality score were 94.58% and 36.55 for all reads, respectively. This data will be useful to prepare molecular markers for breeding of new cultivars having a crisp texture and feel because the flesh of the Japanese pear has many stone cells [4]. Additionally, this data will also be valuable for a more detailed study of the evolutionary processes occurring in *Pyrus* species by providing genomic data for a comparative analysis with Chinese pear [5] and European pear [6] that have already been analyzed.

## 2 Experimental design, materials, and methods

### 2.1 DNA extraction

Genomic DNA was extracted from the young leaves of the Japanese pear strain TH3, using a modified version of the cetyltrimethylammonium bromide (CTAB) protocol [7]. Quality and quantity of DNA were checked by Agilent 2200 TapeStation (Agilent Technologies, Santa Clara, USA).

### 2.2 Sequencing

The extracted genomic DNA was subjected to preparation of a paired-end library for genome sequencing using the Illumina HiSeq. 2500. Genomic DNA libraries were constructed using the TruSeq Nano DNA Library Prep Kit (Illumina, Cat FC-121-4001) according to the manufacturer׳s protocol.

### 2.3 Data accessibility

The read data were submitted to the DDBJ Read Archive (BioProject: PRJDB6878, BioSample: SAMD00117051).
